# The Medical Corps of the Navy

**Published:** 1888-01

**Authors:** 


### THE MEDICAL CORPS OF THE NAVY.

We are glad to observe that the attention of Congress is likely to be called before long to the necessity of increasing the attractiveness of the naval service for medical men. As matters are now, either the vacancies must go on increasing in number or the standard of requirements must be lowered to let in an inferior class of men. Whichever of these results ensues, the navy must suffer in consequence.—*New York Medical Journal.*

